# Gelseminum for Vesical Gravel

**Published:** 1874-04

**Authors:** 


OUR correspondent, Dr. A. Shamblin, of Broom Town, Ala., writes us that he has been quite successful in the removal of small calculi from the bladder by the use of gelseminum sempervirens, which he exhibits in powder. He states that one of his cases, in the four years past, has voided, in all, *seventeen* stones from his bladder, of varying size, one of which was as large as a small mulberry, and very rough.

Dr. Shamblin employs copious diluent drinks with stimulating diuretics for twelve to fifteen hours, and then directing his patient to retain the urine as long as possible, he administers the powdered gelseminum every two hours until general relaxation occurs, when the patient is placed in the knee-elbow position and directed to void his urine forcibly. His success, in repeated cases, by this method, has been gratifying. The *modus operandi* is obvious, the treatment based upon correct principles, and we doubt not will be found in very many cases successful.

